# HARNESSING ARTS IN MEDICINE FOR STRENGTH IN AGE: CASE STUDY, CENTER FOR PERFORMING ARTS MEDICINE, HOUSTON METHODIST

**DOI:** 10.1093/geroni/igz038.113

**Published:** 2019-11-08

**Authors:** J Todd Frazier

**Affiliations:** Houston Methodist Hospital- Center for Performing Arts Medicine, Houston, Texas, United States

## Abstract

In the 1970’s hybrid arts and humanities programs began developing within healthcare systems across the country. Today, the National Organization for Arts in Health (NOAH) represents a network of hundreds of programs with a major focus of serving older people and their caregivers. This presentation will provide an overview of this rapidly developing network along with a multimedia overview of a comprehensive arts and medicine hospital based program in the Texas Medical Center: Houston Methodist Hospital’s System Center for Performing Arts Medicine one of NOAH’s founding members. The Center’s expanding national, regional and community network of artist health, arts integration, creative arts therapy, research, and outreach programs serving older people will be described. Demonstrating how arts in health communicate and elevate value across disciplines through program evaluation (patient satisfaction and employee opinion), clinical research, and financial and outcome data will be explored as a case study.

